# Retinal Vascular Image Segmentation Using Improved UNet Based on Residual Module

**DOI:** 10.3390/bioengineering10060722

**Published:** 2023-06-14

**Authors:** Ko-Wei Huang, Yao-Ren Yang, Zih-Hao Huang, Yi-Yang Liu, Shih-Hsiung Lee

**Affiliations:** 1 Department of Electrical Engineering, National Kaohsiung University of Science and Technology, Kaohsiung 80778, Taiwan; elone.huang@nkust.edu.tw (K.-W.H.); f109154154@nkust.edu.tw (Y.-R.Y.); f110154124@nkust.edu.tw (Z.-H.H.); I108154101@nkust.edu.tw (Y.-Y.L.); 2 Department of Urology, Kaohsiung Chang Gung Memorial Hospital, Chang Gung University College of Medicine, Kaohsiung 83301, Taiwan; 3 Department of Intelligent Commerce, National Kaohsiung University of Science and Technology, Kaohsiung 82444, Taiwan

**Keywords:** medical image, retinal vessel segmentation, deep learning, neural network

## Abstract

In recent years, deep learning technology for clinical diagnosis has progressed considerably, and the value of medical imaging continues to increase. In the past, clinicians evaluated medical images according to their individual expertise. In contrast, the application of artificial intelligence technology for automatic analysis and diagnostic assistance to support clinicians in evaluating medical information more efficiently has become an important trend. In this study, we propose a machine learning architecture designed to segment images of retinal blood vessels based on an improved U-Net neural network model. The proposed model incorporates a residual module to extract features more effectively, and includes a full-scale skip connection to combine low level details with high-level features at different scales. The results of an experimental evaluation show that the model was able to segment images of retinal vessels accurately. The proposed method also outperformed several existing models on the benchmark datasets DRIVE and ROSE, including U-Net, ResUNet, U-Net3+, ResUNet++, and CaraNet.

## 1. Introduction

Image segmentation is an important topic in image processing and machine vision. These methods classify all pixels in a given image, and have been widely adopted in a variety of applications such as object detection, scene understanding, autonomous vehicles, and medical images. In recent years, significant advances have been achieved in the use of artificial intelligence (AI) technology for image segmentation. Deep learning models are effective in performing segmentation medical images to better understand their content [1], which also supports research on human robot collaboration (HRC) that aims to increase working efficiency and reduce labor costs. The human eye is a critical part of our visual system. For sighted people, vision typically provides more than 70% of the sensory information processed by the brain in everyday life. The fundus of the eye contains multiple anatomical structures, including the retina. Modern imaging techniques can observe the capillary structure of the retina directly in images of the fundus image to study the physiological characteristics of the retina. Performing segmentation on image of retinal blood vessels is the basis of automated analysis methods. The retinal vessels of the fundus are uniquely observable as deep capillaries, and the influence of various diseases on the retinal vessel network can be reflected by segmenting the morphological structure of retinal vessels. However, retinal vessel segmentation involves some notable challenges relating to the contrast between the target blood vessels of the retina and the background, the width of the vessels and their nonuniform variations, and noise generated in the image acquisition process. In the past, oculists directly observed retinal images using the naked eye, which required considerable time and effort. Retinal lesions are complicated and diverse, and the blood vessels in retinal images are interlaced and differ from one another. Fatigue on the part of oculists can also lead to human error [2].

The retina is a membrane that generated nerve signals based on incident light in visual perception. It consists of several main parts, including optical disks, macula, and blood vessels. The task of segmenting retinal blood vessels has attracted attention as a topic of active research, and variations in the shape and central zone of the retina as well as the sizes of different blood vessels have been highlighted as key difficulties. Furthermore, variations in the eye itself, the optic disk, and the fovea interfere with blood vessel segmentation. Only a limited amount of image data are available to train segmentation models, and existing images are largely nonuniform. Many ocular lesions can also change the morphology of the vascular tree. For example, in diabetic retinopathy [3] and hypertensive retinopathy [4], angiorrhea induces many variations around the vascular tree. Thus, the retinal vascular tree is an important biological criterion in the diagnosis of several eye diseases. Patients are screened for these diseases based on changes in vascular morphology, which are also used to diagnose the conditions and evaluate their severity. However, training an algorithm or segmentation model identifying vascular features intelligently to accurately segment effective target contour structures for applications in clinical diagnosis is difficult given the underlying complexity of the scale, shape, and geometric transformations of the retinal blood vessels.

Several studies have used image processing, computer vision, and machine learning technologies to segment images of retinal blood vessels [5,6,7,8,9,10,11,12,13,14,15,16]. Neural network models have evolved as a mainstream technology and have yielded significant results. Girish et al. [17] proposed a fully convolutional neural network model (FCNN) for intraretinal image segmentation in optical coherence tomography (OCT) images. Despite the simplicity of the model, their experimental results showed that it achieved good results [17]. Park et al. [18] used automatic color equalization (ACE) to preprocess images of retinal vessels and proposed a deep fully convolutional network stacking multiple generative adversarial networks [19] (M-GAN). They also added a multikernel pooling block between the stack layers of the M-GAN model to compensate for variations at different scales. Ye et al. [20] used a fusion neural network to diagnose retinopathy. In their proposed structure, images were segmented using U-Net [21], and quantitative analysis was performed while the data were processed with a ResNet-18 model [22] to output the results for further feature extraction. The results were then merged to predict familial exudative vitreoretinopathy (FEVR). U-Net has been shown to perform well in medical image segmentation. In this study, we propose a structure based on an improved U-Net neural network backbone with an added residual module to enhance the capability of the network to extract features. The proposed method also includes a full-scale skip connection used to combine low-level details with high-level features at different scales. The results of an experimental evaluation of our proposed approach showed that it was able to segment images of retinal blood vessels accurately. In the experiments, we compared the proposed structure with conventional U-Net, ResUNet, U-Net3+, ResUNet++, and CaraNet models on two different benchmark datasets, DRIVE and ROSE.

The remainder of this paper is organized as follows. We briefly review the relevant literature in Section 2. The proposed neural architecture is described in Section 3. In Section 4, we discuss the experimental results and compare the performance of our approach with that of existing methods. Section 5 concludes by summarizing our findings.

## 2. Related Works

### 2.1. U-Net, Residual Module and Inception Block

Ronneberger et al. [21] proposed the U-Net neural network model in 2015. U-Net was used for image segmentation with an encoder–decoder structure. The name of the model refers to its U-shaped architecture, which is divided into two fully convolutional networks. One of the two networks is an encoder, which uses the contracting path to perform feature extraction through downsampling. The model obtains feature maps of different scales through downsampling, and outputs a 32 × 32 feature map from the last layer. The second network is a decoder, which merges the expansive path with the corresponding scale feature map using an overlapping-tile strategy after performing upsampling and feature extraction. U-Net is designed to overcome feature loss occurring during feature transfer in the decoder, and it can also handle the problem of multiscale features and has enhanced feature reservation capabilities. Since its introduction, many studies have been conducted with U-Net structures [23,24,25,26,27,28]. Given these advantages, the proposed approach is based on a U-Net backbone network.

The ResNet model won the ImageNet Large-Scale Visual Recognition Challenge (ILSVRC) in 2015 [22]. ResNet adds residual learning to a neural network model to address the vanishing gradient problem. Although this issue can be mitigated by batch normalization, training accuracy degrades with deeper neural networks. Residual learning adds a shortcut to the convolutional layer, known as a residual block. In addition to compensating for the vanishing gradient problem, it also preserves training accuracy for deeper models. Residual learning is referred to as a plain network or residual block in ResNet. These residual blocks have subsequently been used in several neural network models [29,30]. Following these works, the proposed approach includes residual modules added to the baseline U-Net model for improved feature extraction capability.

One common method to increase the accuracy of neural networks is to simply increase their depth or width for a greater probability of determining optimum parameters. However, this method suffers from problems with overfitting owing to an excessive number of parameters. Therefore, methods to implement deeper or broader neural networks are important. GoogLeNet was the primary neural network model used in the ImageNet Large-Scale Visual Recognition Challenge (ILSVRC) in 2014 [31]. GoogLeNet supports wider and deeper neural networks by incorporating a stack of inception modules, which enables it to extract different features through multiple convolution kernels while changing the width of the neural network. Wider GoogLeNet models require significantly less training, and have correspondingly lower computational complexity. These advantages mitigate the overfitting problem that affected prior neural network architectures. In this study, our proposed network architectures incorporates an inception block to extract more diverse features.

### 2.2. Medical Image Segmentation Methods Based on Deep Learning

The original U-Net performs multiscale feature extraction through up- and downsampling. However, feature information may be lost during this process, and the details of the low- and high-level feature maps are not fully used. U-Net 3+ [32] was proposed to solve this problem. U-Net3+ is based on U-Net. It incorporates a full-scale dense skip connection mechanism and fuses low-level feature details and high-level feature semantics in full-scale feature mapping. Additionally, three loss functions are used, including focal [33], multiscale structural similarity index (MS-SSIM) [34], and intersection over union loss (IOU loss) [35]. As a result of this improved architecture, U-Net3+ is very effective in enhancing the boundaries of organs and reducing oversegmentation in non-organ images. In this study, we used the full-scale skip connection of UNet3+ to combine low-level details with high-level features at different scales to accurately segment retinal vessels. We compared the performance of a U-Net 3+ model with that of the proposed approach in our experimental evaluation, and the results showed that our method performed significantly better on the two public datasets.

In 2019, Pan et al. [36] proposed a retinal vessel segmentation method known as ResUNet to improve U-Net models. ResUNet introduces residual modules to replace convolution operations to reduce the performance degradation associated with deeper networks. ResUNet was verified using the public DRIVE dataset, and its segmentation accuracy was 96.5%. However, ResUNet still loses some feature information and incompletely utilizes the information it does retain. ResUNet++ was proposed as an improved version of ResUNet. It incorporates an attention mechanism [37], channel-wise SENet blocks [38], and atrous spatial pyramid pooling (ASPP) [39] to enhance its receptive field and feature learning capabilities. ResUNet++ also uses a conditional random field (CRF) and test time augmentation (TTA) for better prediction efficiency. Along these lines, many studies have been conducted on medical image segmentation based on deep learning [40,41,42,43,44,45]. In the present work, the proposed structure is designed to improve a U-Net model by adding residual modules. A U-Net backbone and residual modules are adopted to enhance the capability of the network to extract features. It also uses a full-scale skip connection to combine low-level details with high-level features at different scales. More diverse features can be extracted by using inception blocks. The results of our experimental evaluation show that the proposed method outperformed several existing methods, including U-Net, ResUNet, U-Net3+, ResUNet++, and CaraNet, on the public datasets DRIVE and ROSE.

## 3. Proposed Method

**Figure 1:**
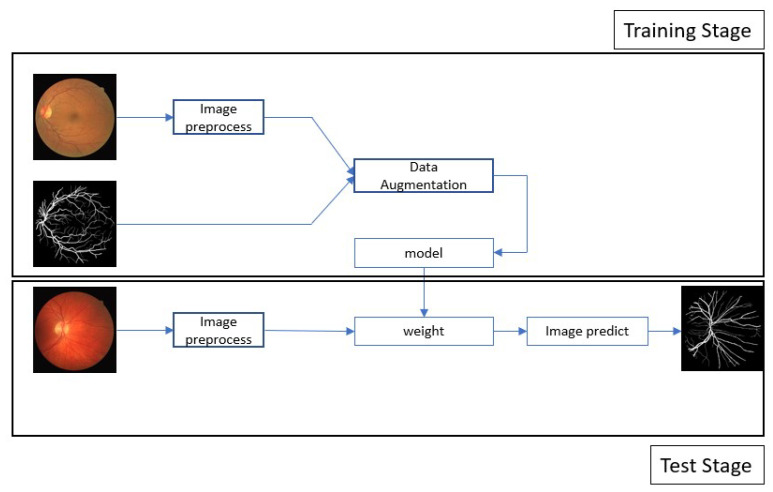
The proposed system architecture.

The proposed neural architecture is based on an improved U-Net model with added residual modules as part of a retinal vessel segmentation system. To evaluate the effectiveness of our method, we trained the model to perform the specified task and tested it experimentally. First, image data were preprocessed and augmented to enhance the diversity of the images, and then training was performed to obtain the parameter weights. Finally, the model was tested and analyzed using the testing images. The entire process is illustrated in Figure 1.

### 3.1. Data Preprocessing

**Figure 2:**
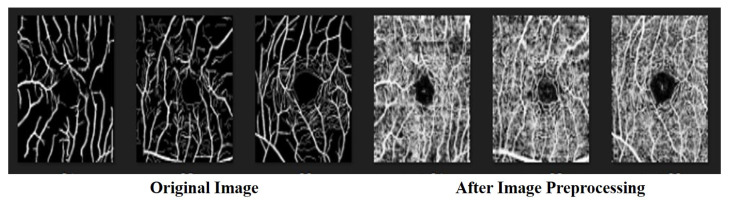
The image preprocessing on ROSE dataset.

**Figure 3:**
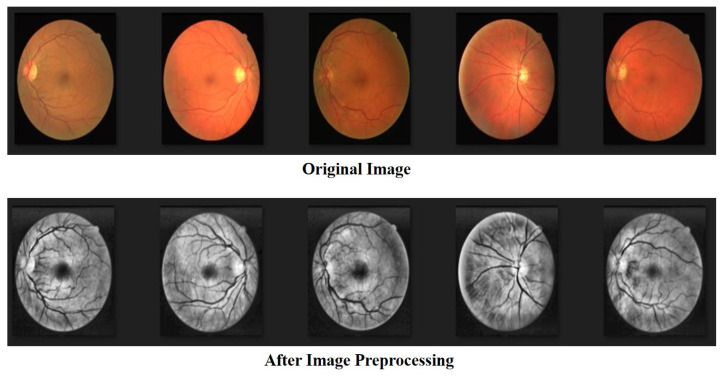
The image preprocessing on DRIVE dataset.

In image recognition, data preprocessing can reduce noise and enhance important features for improved accuracy when performing feature extraction. In this study, all the data were transformed into grayscale images in advance. The color intensity and depth distance in the images were determined using a nonlinear bilateral filter [46] to maintain edges, reduce noise, and apply smoothing. After denoising, we applied contrast-limited AHE (CLAHE) [47] to enhance the contrast between the blood vessels and the background. CLAHE is effective for images with excessively dark or bright backgrounds. In the final step, we performed gamma correction [48] to avoid losing details after brightness control. The process flow used in preprocessing the data is illustrated in Figure 2 and Figure 3.

### 3.2. Model Architecture

**Figure 4:**
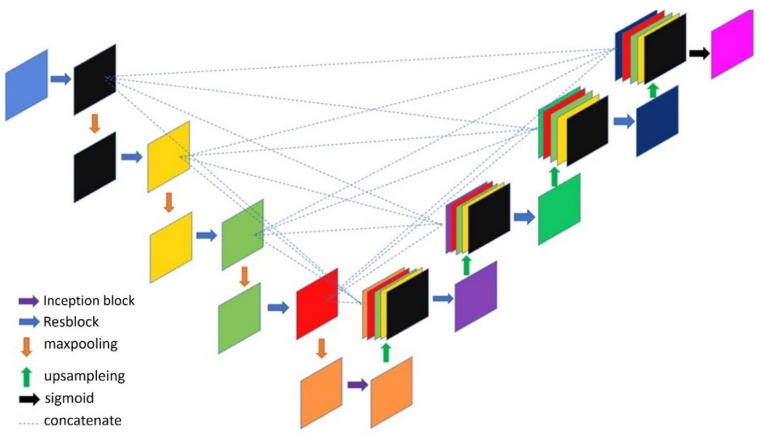
The proposed model architecture.

The proposed architecture based on a U-Net backbone with residual blocks was further optimized for better performance. The general convolution operation in the encoder and decoder was changed to a residual block to enhance the models’ capability to extract features and prevent avoid the vanishing gradient problem. Additionally, the structure includes a full-scale skip connection between the encoder and decoder of the U-Net model to integrate feature information at different scales into the upsampling and downsampling processes to provides finer information during the final image generation. Finally, an inception block is used in the bottleneck feature (or latent representation) of the U-Net structure to allow the feature map to perform convolution operations through convolution kernels of different sizes and allow the model to extract more diverse features. The proposed architecture is illustrated in Figure 4.

### 3.3. Residual Block

**Figure 5:**
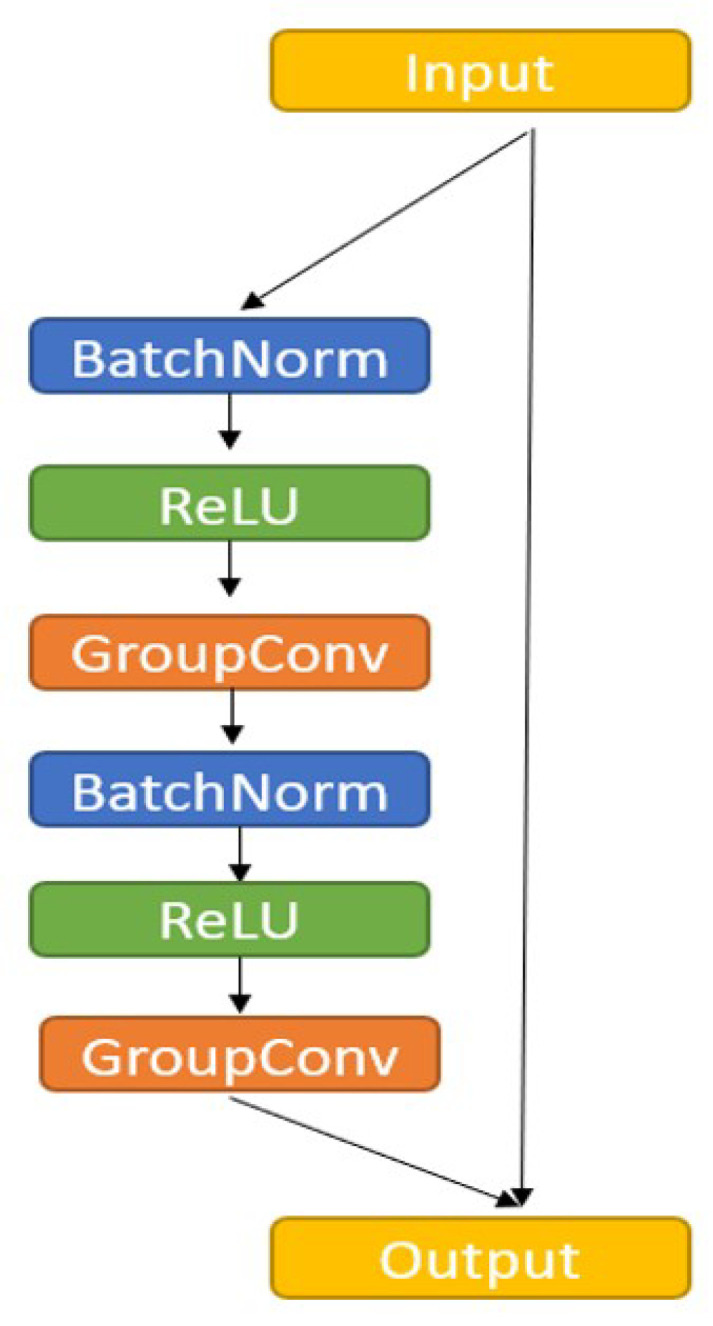
The residual block used in proposed model.

Increasing the depth of a network is key to optimizing performance; however, as the network deepens, vanishing and exploding gradients degrade the effectiveness of the training process. Therefore, we replaced the general convolution layer in U-NET with a ResBlock to enhance the feature extraction capabilities of the model. An identification mechanism is also included with the stack of residual blocks to avoid performance degradation with deeper layers. Additionally, batch normalization is performed in the residual block to mitigate the vanishing gradient problem and accelerate the convergence. We also used the rectified linear unit activation function in each network layer. The residual block used in the proposed model is shown in Figure 5.

### 3.4. Skip Connection

**Figure 6:**
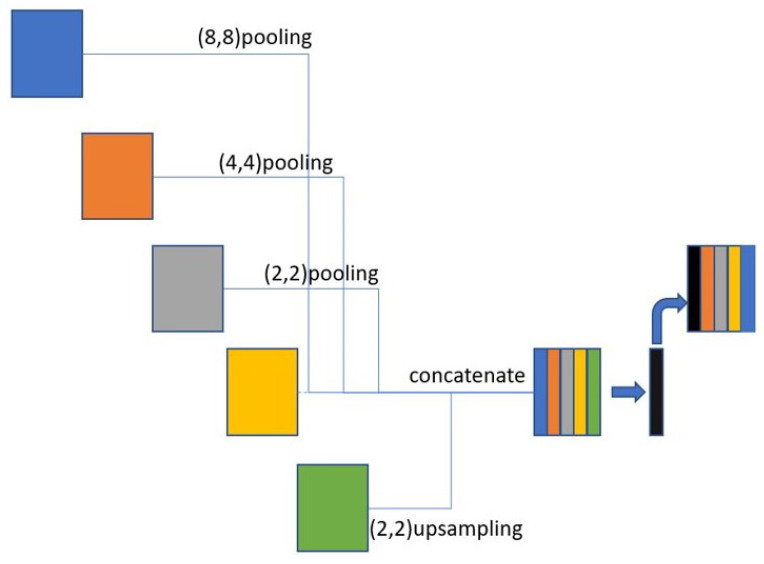
The skip connection used in the proposed model.

In the upsampling and downsampling processes of U-NET, small-scale feature maps are finer but smaller, whereas large-scale feature maps are larger and the features are relatively rough. Therefore, establishing different scales for sharing information is important. U-NET has a skip connection mechanism, but does not directly extract sufficient information from different feature scales. In contrast, our proposed method uses a full-scale skip connection to combine different scales of feature maps to enable the model to refer to features at different scales. For large-scale feature maps, downsampling is performed to unify the sizes of the feature maps, and eight-fold, four-fold, and two-fold downsampling is performed individually. For small-scale feature maps, two-fold upsampling is performed to unify the feature map size. Finally, various feature maps are combined and the features extracted using the residual block. This procedure is repeated as shown in Figure 6.

### 3.5. Inception Block

**Figure 7:**
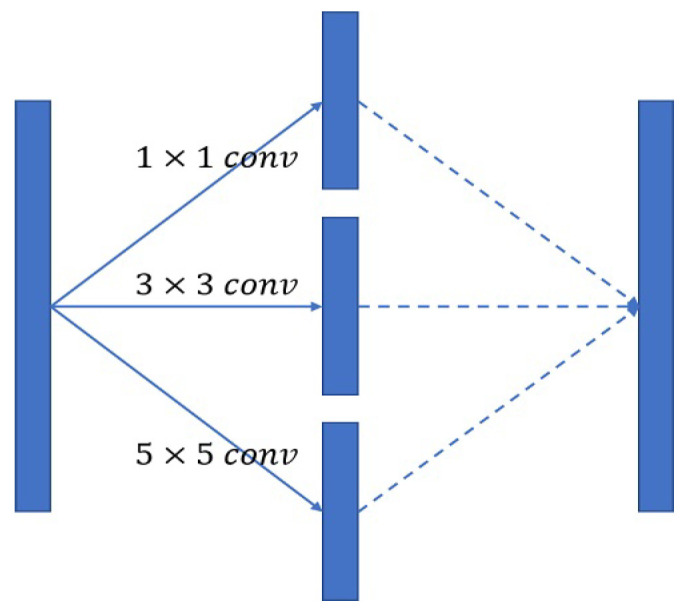
The inception block used in proposed model.

A convolution kernel defines the dimensional range of a convolution and represents the magnitude of visual field sensitivity in a network. A 3 × 3 convolution kernel is most commonly used. Following the inception model used in GoogLeNet, we used 1 × 1, 3 × 3, and 5 × 5 convolution kernels individually in the bottleneck features of the U-Net structure, which were combined to increase the sensitivity of the visual field the diversity of the extracted features, as shown in Figure 7.

## 4. Experiment

We conducted the experimental evaluation with a workstation running the Windows 10 operating system, with an Intel Core i7-10700 CPU with a clock frequency of 2.90 GHz, 32 GB of RAM, and an Nvidia GeForce RTX3070 GPU with 8GB of video memory. The software was written in the Python 3.7 programming language with the Anaconda 3 data science platform. The neural networks were trained using the TensorFlow framework with GPU acceleration and the Keras software (https://keras.io/, accessed on 4 May 2023) library to implement the deep learning models.

### 4.1. Dataset

#### 4.1.1. DRIVE: Digital Retinal Images for Vessel Extraction

**Figure 8:**
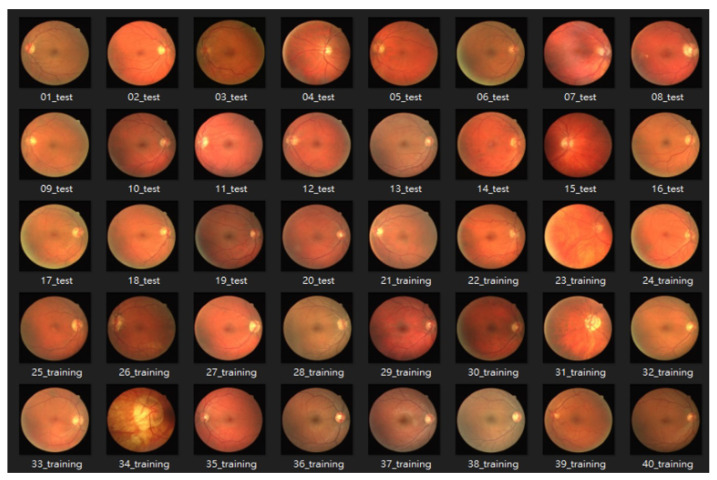
The dataset of DRIVE.

**Figure 9:**
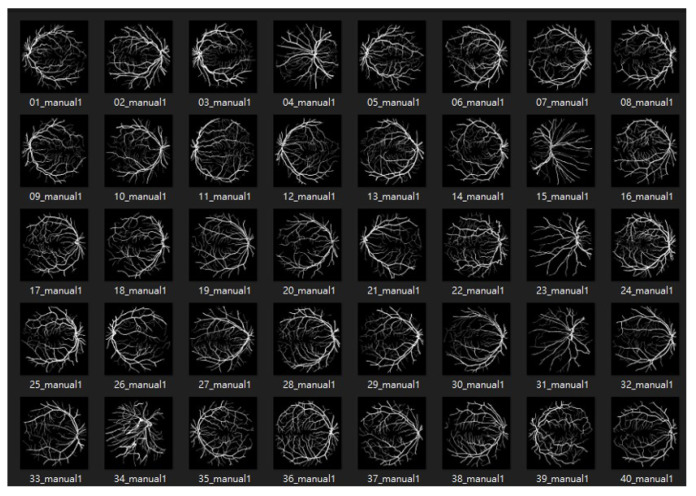
The labeled dataset of DRIVE.

The DRIVE database [49] was created to compare and study retinal vessel segmentation methods. Physicians can use DRIVE to observe the vascular morphology of the retina such as its length, width, sinuosity, and angle to diagnose cardiovascular and ocular diseases including diabetes and hypertension. The photographs provided in the DRIVE database were obtained from a diabetic retinopathy screening project in the Netherlands. The screened participants included 400 diabetes subjects aged 25–90 y. Forty photos were randomly selected, including 20 for the training set and 20 for the testing set, as shown in Figure 8 and Figure 9.

#### 4.1.2. ROSE: A Retinal OCT-Angiography Vessel Segmentation Dataset

**Figure 10:**
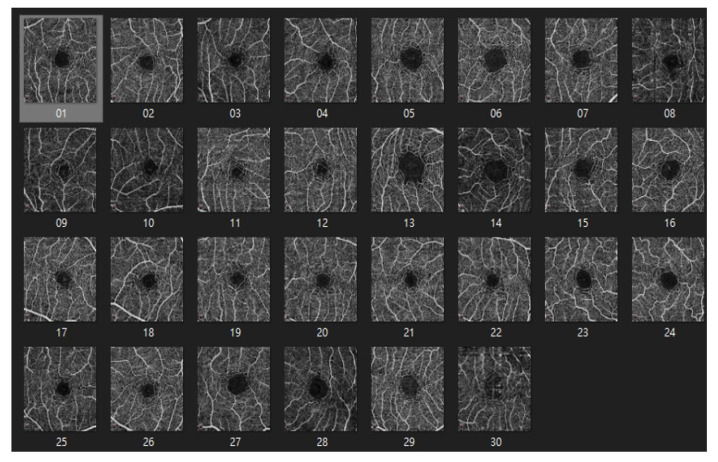
The dataset of ROSE.

**Figure 11:**
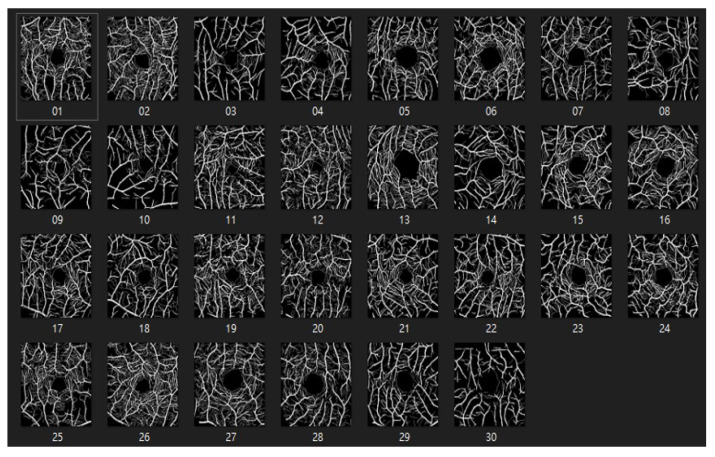
The labeled dataset of ROSE.

The ROSE dataset [50] consists of 39 subjects, including 30 images for training and 9 images for testing. Each image is 304 × 304 pixels in size. Manual annotations of the blood vessels were performed by medical imaging experts and clinicians [51] as shown in Figure 10 and Figure 11.

(1) $$\begin{array}{c} Accuracy=\frac{TP+TN}{TP+FP+FN+TN} \end{array}$$

(2) $$\begin{array}{c} Precision=\frac{TP}{TP+FP} \end{array}$$

(3) $$\begin{array}{c} Recall=\frac{TP}{TP+FN} \end{array}$$

(4) $$\begin{array}{c} F_{1}=2\times \frac{Precision\times Recall}{Precision+Recall} \end{array}$$

(5) $$\begin{array}{c} IOU=\frac{TP}{TP+FP+FN} \end{array}$$

### 4.2. Data Augmentation

The data were augmented before training to increase their diversity [52]. The original images were rotated at random angles, moved, and turned in horizontal and vertical directions. We augmented the dataset to a total of 2000, 5000, and 10,000 images and compared the results to determine the robustness of the model.

### 4.3. Evaluation Indexes

Accuracy, precision, and recall are often used to evaluate the effectiveness of models in classification tasks, as expressed in Equations (1)–(3). In this study, we considered image segmentation was regarded as a classification task in which each pixel was identified as to whether it indicates a blood vessel. We also calculated the F measure, which incorporates both precision and recall, to evaluate the quality of the classification model as expressed in Equation (4). F1-measure is a special form of F measure with a beta value of 1, indicating that both precision and recall are important. Higher F1-values indicate better performance. Finally, we calculated the intersection-over-union (IOU) indices to evaluate the segmentation efficiency of the models as expressed by Equation (5).

### 4.4. Model Effectiveness Evaluation

**Figure 12:**
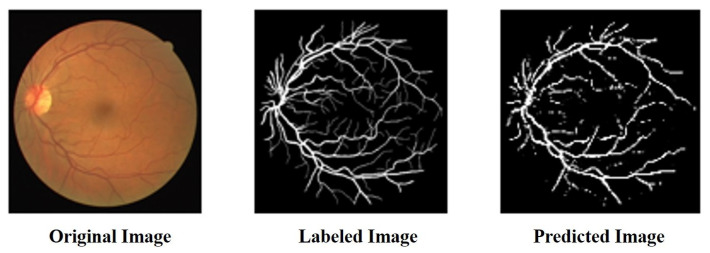
The result of segmentation on DRIVE dataset.

**Figure 13:**
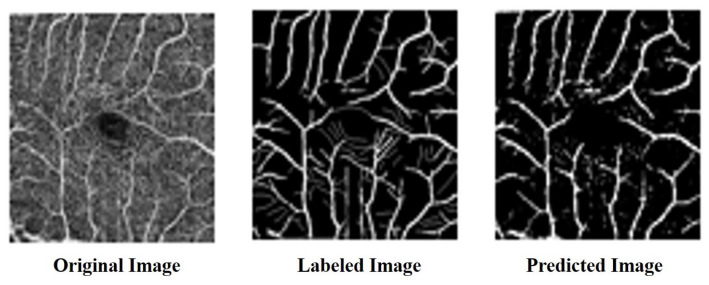
The result of segmentation on ROSE dataset.

**Figure 14:**
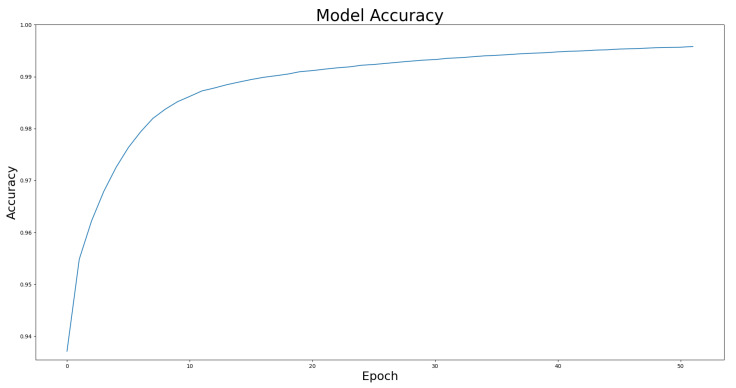
The training curve of accuracy on DRIVE dataset.

**Figure 15:**
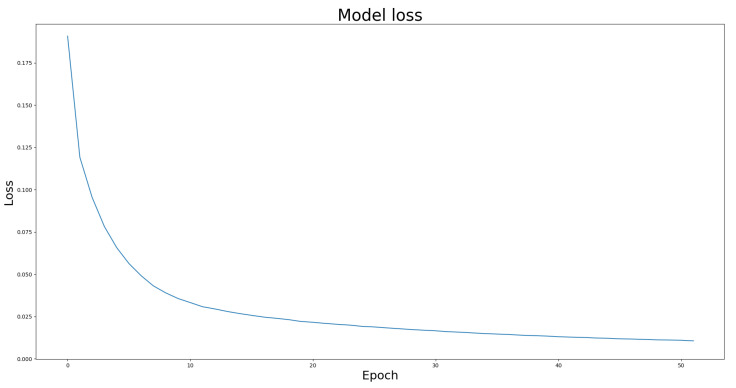
The training curve of loss value on DRIVE dataset.

**Figure 16:**
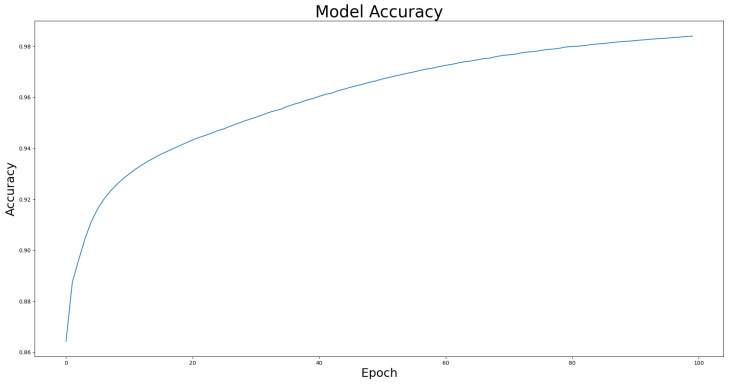
The training curve of accuracy on ROSE dataset.

**Figure 17:**
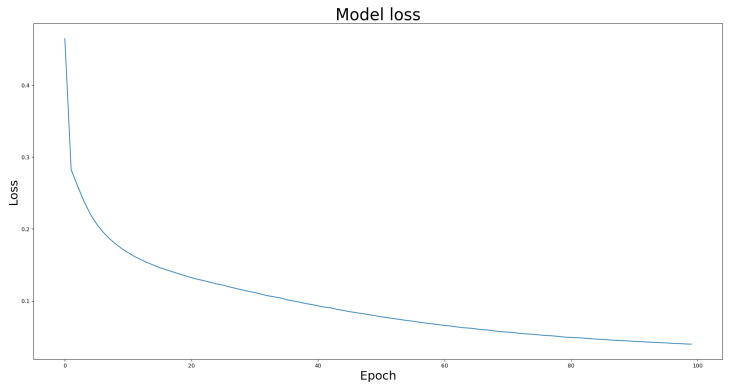
The training curve of loss value on ROSE dataset.

We compared the performance of the proposed architecture with that of U-Net, ResUNet, U-Net3+, and ResUNet++ models individually on the two benchmark datasets DRIVE and ROSE, and the experimental results are listed in Table 1 and Table 2. The data in Table 1 and Table 2 show the best results for various models in the experiments. The experimental results showed that the proposed improved U-NET model with residual blocks, full-scale skip connection, and an inception block performed well. The image segmentation results are presented in Figure 12 and Figure 13. The training curves of the accuracy and loss values are shown in Figure 14, Figure 15, Figure 16 and Figure 17. The training dataset consisted of 5000 samples with a step size of 100, and all the models were trained for 100 epochs. Our proposed method exhibited an accuracy 0.998, while the value of the loss function was 0.003.

### 4.5. Ablation Experiment-Image Preprocessing

The model data before image preprocessing in DRIVE are listed in Table 3. The proposed model achieved the best results for each evaluation index and exhibited good robustness. After preprocessing the images of the DRIVE dataset, the contrast between the blood vessels and the background was increased effectively, and the model was able to extract features more easily. The F1-measure was higher than 70%, which shows that preprocessing the data increased the recognition accuracy of the model. Our proposed method exhibited the highest accuracy (77.8%), as shown in Table 3.

After pre-processing the ROSE dataset, the blood vessels were not evident in the image or were blurred into the background. Because the model could not capture vascular features, it could not perform an accurate classification, as shown in Figure 2. Because preprocessing the data did not improve accuracy, we did not use the preprocessed version of the ROSE dataset in the experimental comparison.

### 4.6. Ablation Experiment-Data Augmentation

The effectiveness of the model when the data were augmented to 2000, 5000, and 10,000 images was compared using the DRIVE dataset. The results showed that the proposed model had the best effect when the data were augmented to 5000 images, with the F1-measure reaching 77.8% as listed in Table 4. We also observed that the proposed structure learned to determine adequately features and performed effectively without requiring large amounts of data.

The effectiveness of the model when the data were augmented to 2000, 5000, and 10,000 images was compared using the ROSE dataset. The results showed that the proposed model exhibited the best performance when the data were augmented to 10,000 images, and the F1-measure reached 74.4% as listed in Table 5.

### 4.7. Compare with State-of-the-Art Model

The proposed model was compared with CaraNet [53], which has recently shown good performance on small-object-segmentation tasks. CaraNet was designed for polyp segmentation and uses a Res2Net [54] architecture with a Channel-wise Feature Pyramid (CFP) [55] module to obtain multiscale high-level features. It also establishes the relationship between global contours and high-level features using a reverse attention module. As shown in Table 6, the performance of CaraNet was significantly inferior to that of the proposed model because it processes only high-level features, which easily leads to the loss of the fine counters of blood vessels. In contrast, the proposed model did not lose the features of fine blood vessels and performs better in applications requiring precise segmentation owing to its use of skip connections.

## 5. Conclusions

Segmenting the retinal vascular tree is a key step in the detection and diagnosis of various ocular lesions, including diabetic retinopathy, age-related macular degeneration, and glaucoma. In this study, we have proposed an improved U-Net neural architecture that incorporates residual modules. The experimental results demonstrate that our method can save time and reduce misrecognition induced by fatigue in the diagnosis of eye diseases by accurately performing retinal vessel segmentation. Our proposed architecture is based on a U-Net backbone, and the added residual modules improve the feature extraction capabilities of the network. A full-scale skip connection is also used to combine low-level details with high-level features at different scales. Our experimental results show that the proposed approach was able to segment retinal blood vessels accurately. Medical retinopathy imaging data from the DRIVE and ROSE datasets were preprocessed for the evaluation. The results show that the proposed method outperformed conventional U-Net, ResUNet, U-Net3+, ResUNet++, and CaraNet models at the segmentation task on these datasets.

## Figures and Tables

**Table 1:** Comparison of models on DRIVE dataset.

	Accuracy (%)	Precision (%)	Recall (%)	F1-Score (%)	IOU (%)
U-NET	**97.5**	69.3	**86.7**	**76.9**	58.5
ResUNet	97.3	68.3	85.6	75.3	57.9
UNET3+	97.3	70.6	**89.7**	76	58.1
ResUNet++	**97.6**	**73.1**	85.8	76.8	**59.8**
proposed model	**97.5**	**73.1**	85.4	**77.8**	**60.8**

**Table 2:** Comparison of models on ROSE dataset.

	Accuracy (%)	Precision (%)	Recall (%)	F1-Score (%)	IOU (%)
U-NET	94.2	66.1	**94.1**	73	**58.8**
ResUNet	94.2	63.1	**88.4**	72.7	57.2
UNET3+	94.1	66.3	**88.4**	73.2	58.5
ResUNet++	**94.5**	**67.2**	77.8	**74.8**	**58.8**
proposed model	**95**	**72.3**	80.3	**74.4**	**59.3**

**Table 3:** The result of ablation experiment for image preprocessing on DRIVE dataset.

		Accuracy (%)	Precision (%)	Recall (%)	F1-Score (%)	IOU (%)
U-NET	w/o	96.4	52.8	81.8	64.1	47.2
	w/	**97.5**	69.3	86.4	76.9	58.5
ResUNet	w/o	96.1	51.6	77.1	51.6	44
	w/	97.3	68.3	83.9	75.3	57.9
UNET3+	w/o	96	50.2	77.1	60.8	43.7
	w/	97.3	70.6	83.4	75.8	58.1
ResUNet++	w/o	96.5	58.2	**88.1**	69.5	51.5
	w/	**97.6**	**71.9**	85.8	**77.5**	**59.8**
proposed model	w/o	96.7	60.7	**88.9**	72.2	56.5
	w/	**97.5**	**73.1**	85.4	**77.8**	**60.8**

**Table 4:** The result of ablation experiment for data augmentation on DRIVE dataset.

		Accuracy (%)	Precision (%)	Recall (%)	F1-Score (%)	IOU (%)
U-NET	2000	97.3	69.2	84.1	75.9	58.2
	5000	97.4	68.3	**86.7**	76.4	58.4
	10,000	**97.5**	69.3	86.4	76.9	58.5
ResUNet	2000	97.1	64.8	83.8	73.7	57.3
	5000	97.3	66.3	85.6	74.7	57.4
	10,000	97.3	68.3	83.9	75.3	57.9
UNET3+	2000	96.7	60.5	**89.7**	72.2	56.8
	5000	97.3	70.5	82.6	76	57.8
	10,000	97.3	70.6	83.4	75.8	58.1
ResUNet++	2000	96.8	68.5	83.5	75.8	59.1
	5000	97.3	69.6	84.9	76.8	59.5
	10,000	**97.6**	71.9	85.8	77.5	59.8
proposed model	2000	97.3	69.1	84.4	76	**60.5**
	5000	**97.5**	**71.5**	85.4	**77.8**	**60.8**
	10,000	97.4	**73.1**	85.8	**77.7**	**60.8**

**Table 5:** The result of the ablation experiment for data augmentation on ROSE dataset.

		Accuracy (%)	Precision (%)	Recall (%)	F1-Score (%)	IOU (%)
U-NET	2000	93.5	58.3	**89.9**	70.7	57.2
	5000	94.1	64.5	**94.1**	72.9	57.4
	10,000	94.2	66.1	82.4	73	**58.8**
ResUNet	2000	94.1	60.2	88.4	71.6	55.8
	5000	94.1	62.3	85.8	72.2	56.5
	10,000	94.2	63.1	85.8	72.7	57.2
UNET3+	2000	94.1	63.1	88.4	72.6	58.5
	5000	94	63.9	83.1	72.3	58.4
	10,000	94	66.3	81.7	73.2	58
ResUNet++	2000	94.2	65.3	76.5	72.5	57.3
	5000	94.2	65.8	77.3	73.1	58
	10,000	94.5	67.2	77.8	**74.8**	**58.8**
proposed model	2000	**94.7**	**70**	77.7	73.7	**59.3**
	5000	**94.7**	**72.3**	76.2	**74.2**	57.6
	10,000	**95**	69.4	80.3	74.4	**59.3**

**Table 6:** The comparison of state-of-the-art model.

	Dataset (%)	Accuracy (%)	Precision (%)	Recall (%)	F1-Score (%)	IOU (%)
Proposed model	Drive	**97.5**	**73.1**	85.4	**64.1**	**60.8**
	Rose	**95**	**72.3**	80.3	**76.9**	**59.3**
CaraNet	Drive	70.3	40.3	**96.8**	56.9	28.4
	Rose	59.5	41.7	**99**	58.6	29.3

## Data Availability

The dataset is available for download at https://paperswithcode.com/dataset/rose and https://paperswithcode.com/dataset/drive (accessed on 28 January 2023).
